# The long noncoding RNA HOTAIRM1 controlled by AML1 enhances glucocorticoid resistance by activating RHOA/ROCK1 pathway through suppressing ARHGAP18

**DOI:** 10.1038/s41419-021-03982-4

**Published:** 2021-07-14

**Authors:** Liang Liang, Wenbin Gu, Meng Li, Ran Gao, Xin Zhang, Chongye Guo, Shuangli Mi

**Affiliations:** 1 Key Laboratory of Genomic and Precision Medicine, Beijing Institute of Genomics, Chinese Academy of Sciences, China National Center for Bioinformation, Beijing, China; 2 University of Chinese Academy of Sciences, Beijing, China

**Keywords:** Phylogenomics, Mechanisms of disease, Drug regulation, Non-coding RNAs, Leukaemia

## Abstract

Acquired resistance to glucocorticoids (GCs) is an obstacle to the effective treatment of leukemia, but the molecular mechanisms of steroid insensitivity have not been fully elucidated. In this study, we established an acquired GC-resistant leukemia cell model and found a long noncoding RNA, HOTAIRM1, was overexpressed in the resistant cells by transcriptional profiling, and was higher expressed in patients with poor prognosis. The whole-genome-binding sites of HOTAIRM1 were determined by ChIRP-seq (chromatin isolation by RNA purification combined with sequencing) analysis. Further study determined that HOTAIRM1 bound to the transcriptional inhibitory region of ARHGAP18 and repressed the expression of ARHGAP18, which led to the increase of RHOA/ROCK1 signaling pathway and promoted GC resistance through antiapoptosis of leukemia cells. The inhibition of ROCK1 in GC-resistant cells could restore GCs responsiveness. In addition, HOTAIRM1 could also act as a protein sequester to prevent transcription factor AML1(acute myeloid leukemia 1) from binding to the regulatory region of ARHGAP18 by interacting with AML1. At last, we also proved AML1 could directly activate the expression of HOTAIRM1 through binding to the promoter of HOTAIRM1, which enriched the knowledge on the regulation of lncRNAs. This study revealed epigenetic causes of glucocorticoid resistance from the perspective of lncRNA, and laid a foundation for the optimization of glucocorticoid-based leukemia treatment strategy in clinic.

## Introduction

Glucocorticoids (GCs) are steroid hormones with anti-inflammatory and immunosuppressive effects [1–4]. Synthetic glucocorticoid analogues, such as dexamethasone (Dex) and prednisolone, are usually used in combination with other chemotherapy drugs for the treatment of hematological malignancies because they can induce apoptosis of lymphocytic cells [5–8]. Unfortunately, glucocorticoid resistance is the main cause of treatment failure [9–11]. It can be induced by a variety of mechanisms [12, 13], which may be different in individuals [14]. For example, the decrease in corticosteroid responsiveness may be attributed to the decrease in number of GRs (glucocorticoid receptors) [15], the change in affinity of GRs ligands [16], the decrease in binding ability of GRs to DNA [17], or the increase in expression of inflammatory transcription factors (such as AP-1) competing for DNA binding [18]. Nevertheless, the understanding of GC resistance is still incomplete.

GCs induce apoptosis by activating intrinsic mitochondrial apoptosis pathway, which is regulated by pro- and antiapoptotic BCL2 family proteins [19]. Some signaling pathways are critical for GC-induced apoptosis [20, 21], and GC resistance is often related to the defect of apoptosis machinery rather than GR [22, 23]. For example, BIM plays an essential role in Dex-induced apoptosis [24]. It was upregulated through p38-MAPK activation in acute lymphoblastic leukemia cells after dexamethasone treatment, which could be blocked by p38-MAPK inhibitor [25]. These facts implicate that the deficiency of apoptosis ability or upstream signal transduction pathways not only provide cancer cells with an intrinsic survival advantage, but also may lead to inherent resistance to chemotherapeutic drugs.

Recently, it has been found that long noncoding RNAs (lncRNAs) can regulate the sensitivity of tumor cells to drugs by participating in cell cycle, apoptosis, DNA damage repair, drug metabolism, and other pathways [26–28]. However, the study of lncRNA on glucocorticoid resistance is very rare. Currently, lncRNA GAS5 has been found to interact with GR [29] and enter the nucleus together, which blocks the binding of GR to GR binding element (GRE) by competing for GR, thus inhibiting the expression of GR target genes [30, 31]. A study has shown that after treatment with methylprednisolone (MP) in vitro, the expression of endogenous GAS5 increased in drug-resistant peripheral blood mononuclear cells. The expression of GAS5 in MP poor responders was higher than that in MP good responders, which indicates that GAS5 may participate in GC resistance [32]. Another lncRNA that may be related to GC resistance is SRA. SRA can interact with the nuclear receptor co-activator SRC-1, which is recruited by nuclear receptors, including PR and GR [33]. In HeLa cells, the genes regulated by GR changed after SRA knockdown, which suggests that SRA may affect GR and then GC resistance [34]. Although lncRNAs GAS5 and SRA have been confirmed to interact with GR or be able to affect the regulation of GR target genes, it is unclear whether they can play a direct role in GC resistance.

The emergence of lncRNAs provides a new field for the study of GC resistance. It will help to understand the molecular mechanism of GC resistance comprehensively, lay the foundation for overcoming the GC resistance and improving the curative efficacy of glucocorticoid drugs.

## Results

### Establishment of glucocorticoid-resistant cells

**Fig. 1:**
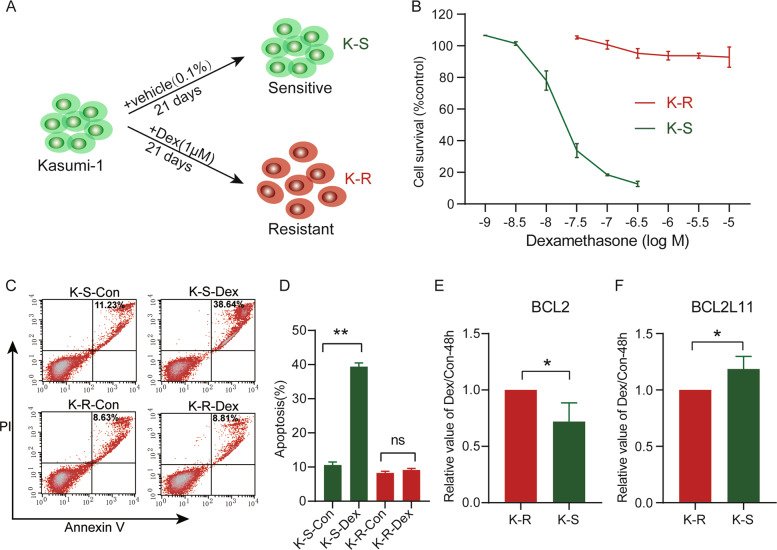
Establishment and characterization of GC-resistant Kasumi-1 cells (K-R). **A** Schematic diagram of the experimental approach for the establishment of glucocorticoid-resistant cells in vitro. In continued presence of 1 µM dexamethasone, the residual survival colonies of Kasumi-1 were isolated and iteratively passaged over 21 days. These colonies were designated as K-R. At the same time, the same operation was performed in the control group treated with the same concentration of vehicle, named K-S. **B** K-R and K-S cells were treated with various concentrations of dexamethasone for 4 days, and the cell viability was measured by MTS assay. **C** Flow cytometry analysis of Annexin V and PI in K-R and K-S cells. Both cells were treated with vehicle and 10 µM dexamethasone for 48 h prior to staining. **D** Quantitative analysis of Annexin V+/PI+ in flow cytometry. **E** Expression ratio of BCL2 in glucocorticoid group and control group after 48 h Dex treatment. **F** Expression ratio of BCL2L11 in glucocorticoid group and control group after 48 h Dex treatment. Data information: Data were represented as mean ± SD, *n* = 3 (**B, D–F**). Difference between groups was assessed using Student’s *t*-test: *p < 0.05, **p < 0.01, ***p < 0.001, ns means no statistical significance.

In order to study the possible causes of GC resistance, we adopted the strategy of inducing sensitive cells into resistant cells to minimize the impact of differences in genetic background between different cells. Kasumi-1, which is a GC-sensitive t(8:21) acute myeloid leukemia cell line [35], was transformed into GC-resistant cells after continuous exposure to 1 μM dexamethasone ethanol solution for 21 days, and designated as Kasumi-1-dexamethasone-resistant cells (K-R). Meanwhile, the corresponding ethanol solvent treatment group was defined as Kasumi-1-dexamethasone-sensitive cells (K-S) (Fig. 1A). The drug sensitivity assay showed dexamethasone could inhibit K-S at low concentrations, while even high concentrations of dexamethasone could not inhibit the growth of K-R (Fig. 1B). K-R cells could maintain GC resistance after 25 days of culture in GC-withdrawn medium (Supplementary Fig. S1A), indicating that the established K-R have stable and long-lasting GC resistance. Morphologically, the distribution of K-R in medium was more dispersed than that of K-S (Supplementary Fig. S1B), suggesting that it may have different characteristics between K-R and K-S. Compared with K-S, the apoptosis rate of K-R treated with GC for 48 h decreased remarkably (Fig. 1C, D). Gene expression detection showed that after 48 h of Dex stimulation, the change ratio of antiapoptotic gene BCL2 in K-R was significantly higher than that of K-S (Fig. 1E), while the change ratio of proapoptotic gene BCL2L11 was significantly lower than that of K-S (Fig. 1F). These results further confirmed that K-R and K-S had significant differences in glucocorticoid resistance.

### HOTAIRM1 enhanced dexamethasone resistance by affecting apoptosis

We first considered the known glucocorticoid resistance mechanisms in this model. There was no gene mutation and copy number change in GR in K-R cells. The expression level of GR protein had no significant difference between K-R and K-S (Fig. S2A, B).

**Fig. 2:**
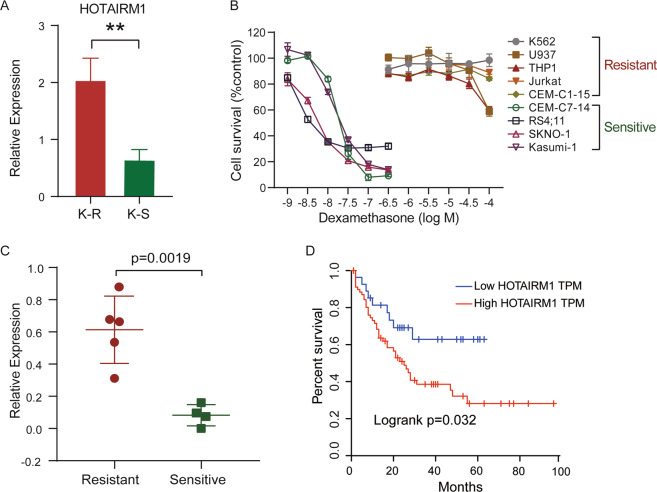
High expression of HOTAIRM1 in GC-resistant leukemia cells. **A** The relative expression of HOTAIRM1 was measured in K-R and K-S using quantitative RT-PCR (qRT-PCR). ACTB was the reference gene. Data represent the mean ± SD of three experiments, *n* = 3. Difference between groups was assessed using Student’s *t-*test: *p < 0.05, **p < 0.01, ***p < 0.001, ns means no statistical significance. **B** Detection of GC sensitivity among leukemia cell lines (K562, U937, THP1, Jurkat, CEM-C1-15, CEM-C7-14, RS4;11, SKNO-1, Kasumi-1). Cells were treated with various concentrations of dexamethasone for 4 days, and the cell viability was determined by MTS assay. Based on the results, these cell lines were divided into two groups: resistant cells and sensitive cells. **C** Relative expression of HOTAIRM1 in corresponding cells with different responses (Resistant and Sensitive) to GCs. Difference between groups was assessed using Student’s *t*-test (*p* = 0.0019). **D** Kaplan–Meier overall survival curve of AML patients according to HOTAIRM1 expression level from TCGA data, *P*-value of log-rank test is 0.032.

Next, we detected the difference in gene expression between sensitive and resistant cells. Through transcriptome analysis, we found 844 genes upregulated and 301 genes downregulated in K-R compared with K-S (Log2 Fold Change > 1 or <−1, *P*-value < 0.05) (Supplementary Table S1). Among the differentially expressed genes, lncRNAs accounted for a certain proportion. HOTAIRM1(HOXA transcript antisense RNA, myeloid-specific 1) was one of the significantly upregulated lncRNAs in K-R (Fig. 2A), which was located in the HOX gene cluster [36–39]. Then, we checked the expression of HOTAIRM1 in nine leukemia cells including K562, U937, THP1, Jurkat, CEM-C1-15, CEM-C7-14, RS4;11, SKNO-1, and Kasumi-1. According to the sensitivity to Dex determined by IC50 (the half-maximal inhibitory concentration) assay, these cell lines were divided into two groups: resistant cells and sensitive cells (Fig. 2B, Supplementary Table S2). We found that the expression of HOTAIRM1 in resistant cells was significantly higher than that in sensitive cells (Fig. 2C). The analysis of the AML data repository from TCGA (The Cancer Genome Atlas) [40] revealed that HOTAIRM1 expression was higher across AML samples than that in paired normal tissues (Supplementary Fig. S2C). Additionally, Kaplan–Meier survival analysis indicated that high expression of HOTAIRM1 in AML patients was associated with poor overall survival and recurrence-free survival (Fig. 2D). These findings led us to further explore the function of HOTAIRM1 in dexamethasone resistance.

**Fig. 3:**
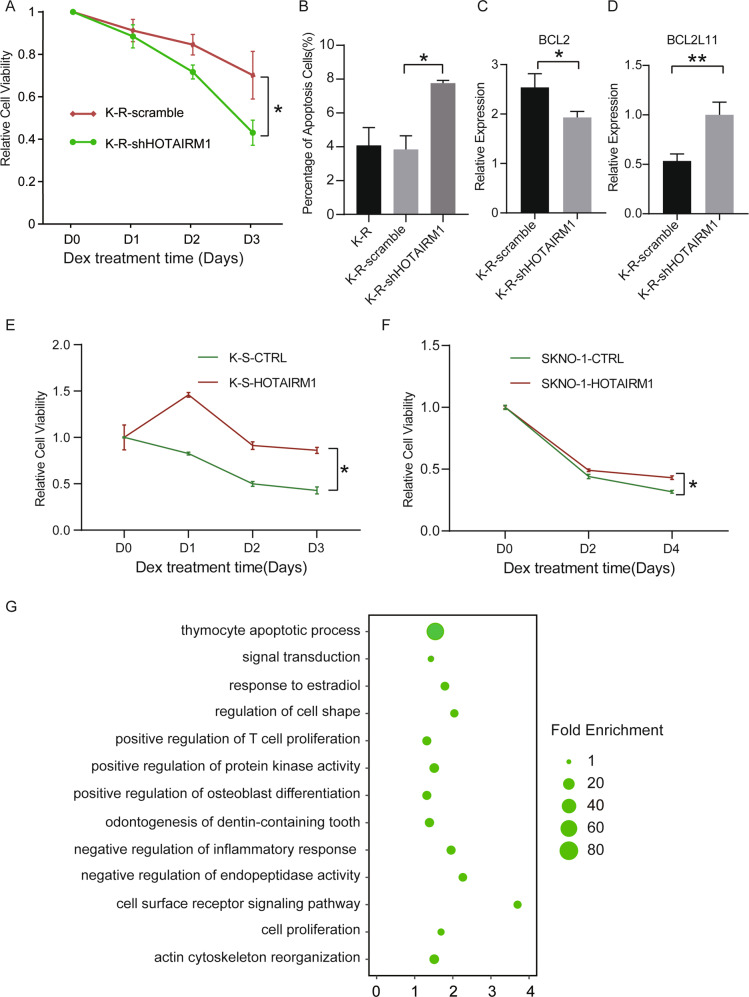
HOTAIRM1 made cells develop dexamethasone resistance by influencing apoptosis. **A** Cell viability rate of control or HOTAIRM1 knockdown K-R cells after dexamethasone (10 µM) treatment from day 0 to day 3. **B** Quantitation of Annexin V+/PI+ in K-R, K-R-scramble or K-R with HOTAIRM1 knockdown cells. All cells were treated with vehicle and 10 µM dexamethasone for 48 h prior to staining. **C** Relative expression of BCL2 in control or HOTAIRM1 knockdown K-R cells. **D** Relative expression of BCL2L11 in control or HOTAIRM1 knockdown K-R cells. **E** Cell viability rate in control or HOTAIRM1 overexpression K-S cells after dexamethasone (10 µM) treatment from day 0 to day 3. **F** Cell survival rate in control or HOTAIRM1 overexpression SKNO-1 cells after dexamethasone (10 µM) treatment from day 0 to day 4. **G** Significantly enriched pathways in genes affected by HOTAIRM1 knockdown in K-R cells (log2 Fold Change > 1 or <−1 and *P*-value < 0.05). The abscissa axis indicates the −log 10 *P-*value, and the ordinate axis indicates Gene Ontology terms. Data information: Data were represented as mean ± SD, *n* = 3 (**A–F**). Difference between groups was assessed using Student’s *t*-test: *p < 0.05, **p < 0.01, ***p < 0.001, ns means no statistical significance.

We knocked down HOTAIRM1 in K-R (Supplementary Fig. S3A) and found the tolerance of cells to glucocorticoids was significantly reduced (Fig. 3A). At 48 h after GC treatment, K-R cells with HOTAIRM1 knockdown showed higher rate of apoptosis compared with control (Fig. 3B). The antiapoptosis gene BCL2 (Fig. 3C) and proapoptosis gene BCL2L11 (Fig. 3D) also changed accordingly. Meanwhile, cell viability was not affected by HOTAIRM1 knockdown without Dex treatment (Supplementary Fig. S3B), indicating that HOTAIRM1 did not affect cell growth in the absence of Dex treatment. On the contrary, overexpression of HOTAIRM1 in K-S and another GC-sensitive cell line SKNO-1 could significantly enhance GC resistance (Fig. 3E, F).

Then we investigated the regulatory relationship between GR and HOTAIRM1. First, we used GR antibody to do ChIP-seq in K-R and K-S, but did not find GR binding in regulator region of HOTAIRM1. Then we detected the protein expression levels after HOTAIRM1 knockdown, and did not observe significant effect on the expression of GR (Supplementary Fig. S3C). Moreover, most of the reported GR target genes were not affected by HOTAIRM1 knockdown (Supplementary Fig. S3D). The above results imply HOTAIRM1 may not affect GC resistance through GR.

To understand the effect of genes regulated by HOTAIRM1 on dexamethasone sensitivity, we conducted transcriptome comparison analysis between K-R and K-R with HOTAIRM1 knockdown (K-R-KD). We obtained 110 differentially expressed genes (Log2 Fold Change > 1 or <−1, *p* < 0.05), among which 74 were upregulated and 36 were downregulated (Supplementary Fig. S3E, Supplementary Table S3). Further functional enrichment analysis showed that the differentially expressed genes were significantly enriched in the pathway of thymocytes apoptotic process (Fig. 3G). These results suggested that HOTAIRM1 regulated cell resistance to glucocorticoids by affecting the apoptosis pathway.

### Genome-wide binding profile of HOTAIRM1 in K-R cells

**Fig. 4:**
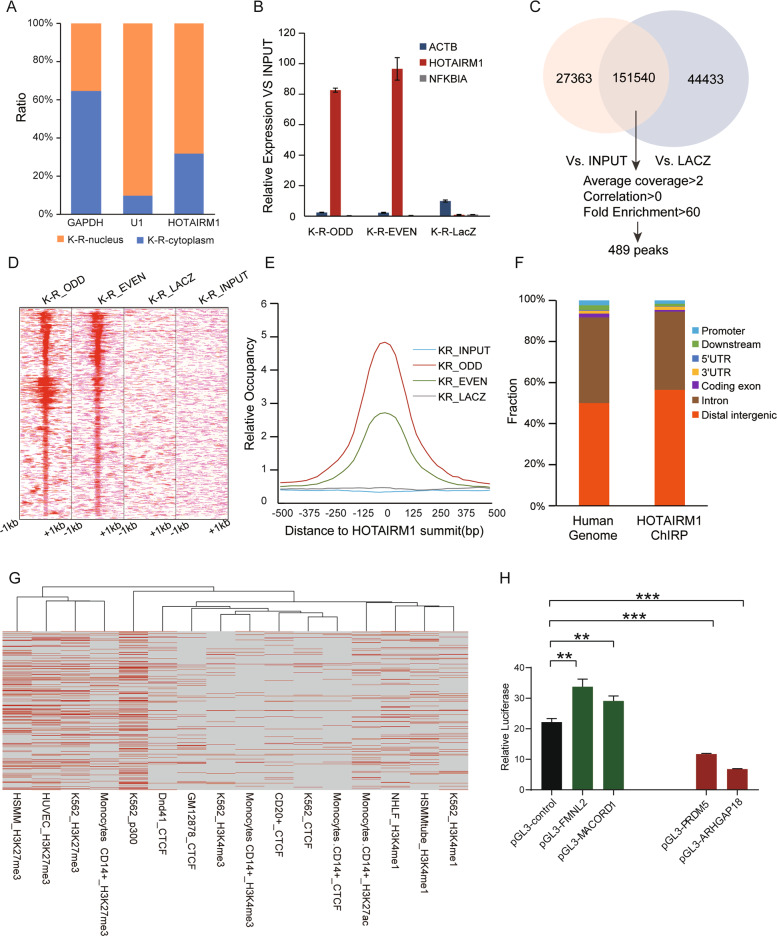
HOTAIRM1 functioned as a *trans*-regulator by binding to genomic DNA. **A** Subcellular fractionation of HOTAIRM1. GAPDH and U1 served as the controls of cytoplasm and nucleus, respectively. **B** HOTIRM1 RNA was enriched specifically by oligo nucleotide probes complementary to HOTAIRM1, compared to the LacZ control. High abundance gene ACTB and medium abundance gene NFKBIA were used as reference in qPCR. **C** HOTAIRM1 binding peaks were called by comparing with the binding sequences of LacZ control or input, respectively. Then 489 peaks were selected by the filtration criteria (Average coverage > 2, Correlation > 0, Fold Enrichment > 60). **D** Heatmap of HOTAIRM1 ChIRP-seq signal in peak regions. Each row represented a 2 kb genomic window centered on a HOTAIRM1 ChIRP peak; the peaks were aligned for HOTAIRM1 binding sites. Red color intensity indicated the number of ChIRP-seq reads. The equivalent genomic windows in control showed that ChIRP with LacZ probes retrieved no signal. **E** Metagene analysis of genomic regions aligned by 489 HOTAIRM1 ChIRP peaks showed focal HOTAIRM1 peaks. **F** HOTAIRM1 binding sites were enriched in distal intergenic region. **G** Hierarchical clustering of the HOTAIRM1 binding peaks (red) distribution on various cis-regulatory elements including repressed autosomal regions marked by H3K27me3, active enhancer-like regions marked by H3K27ac and p300, CTCF binding regions, and promoter-like regions marked by H3K4me3. HSMM means human skeletal muscle myoblasts, HUVEC means human umbilical vein endothelial cells, Dnd41 means acute lymphocyte B cell leukemia cells, NHLF means human normal lung fibroblasts, K562 means myeloid leukemia cells, GM12878 means human B lymphocytes. **H** HOTAIRM1 binding sites acted as transcriptional regulatory elements. K-R cells were transfected with the indicated reporter constructs (Fig. S4C) in luciferase reporter assay. Luciferase activity was compared with that of the empty construct with pGL3 promoter. Data were represented as mean ± SD, *n* = 3. Difference between groups was assessed using Student’s *t*-test: *p < 0.05, **p < 0.01, ***p < 0.001, ns means no statistical significance.

We further studied the regulation mechanism of HOTAIRM1. First, we separated the nucleus and cytoplasm and detected the expression of HOTAIRM1. We found HOTAIRM1 was mainly distributed in the nucleus (Fig. 4A), which ruled out its role as a ceRNA (competing endogenous RNAs). Furthermore, we tested the HOXA family genes close to its genome location, and found that the expression of adjacent HOXA genes were not affected by HOTAIRM1 knockdown (Supplementary Fig. S4A). Therefore, we speculated that HOTAIRM1 may function as a *trans*-regulator.

Next, we adopted ChIRP-seq (chromatin isolation by RNA purification combined with high throughput sequencing) to determine the genome-wide binding sites of HOTAIRM1 [41, 42]. We designed ODD and EVEN probes specifically for HOTAIRM1 isoform 1, because isoform 1 decreased the most when HOTAIRM1 was knocked down (Supplementary Fig. S3A), which may contribute the most to the phenotype. Compared with the LacZ control, HOTAIRM1 probes could strongly enriched HOTAIRM1 in ChIRP assay (Fig. 4B). Among the five isoforms, HOTAIRM1 -1 was significantly more abundant than other transcripts (Supplementary S4B). The genomic DNA fragments interacted with endogenous HOTAIRM1 were sequenced. Using MACS14 paired-end peak caller [43], we identified HOTAIRM1 binding sites and filtered the peaks by average coverage, correlation between odd and even probes, and fold of enrichment to get more reliable binding peaks. Finally, 489 peaks across whole genome were obtained (Fig. 4C, D) and 747 genes were annotated by GREAT (Genomic Regions Enrichment of Annotations Tool) [44] as the target genes for HOTAIRM1 binding (Supplementary Table S4). The focal peak range of HOTAIRM1 binding sites was 200–1000 bp (Fig. 4E), that was similar to the ranges of lncRNAs HOTAIR and TERC binding peaks [41]. These HOTAIRM1 binding sites were widely distributed in the genome, and preferentially occurred in the intron and intergenic regions (Fig. 4F).

We compared the binding sites of HOTAIRM1 with known functional regulatory regions to gain more insight, including various histone modifications (H3K4me3, H3K4me1, H3K27me3, and H3K27ac) and DNA binding proteins (p300, CTCF) in different cells in the ENCODE (Encyclopedia of DNA Elements) database (GEO: GSE29611). We found that the binding sites of HOTAIRM1 had more overlap with the suppression marker H3K27me3 regions, and less intersection with the enhancer marker H3K4me1 and p300 regions (Fig. 4G), indicating that HOTAIRM1 may bind more in gene suppression region and less in activation region. In addition, HOTAIRM1 binding sites only had a little overlap with the H3K4me3 regions, which was consistent with our finding that HOTAIRM1 peaks rarely distributed in promoter region, implying that HOTAIRM1 may not play a regulatory role in promoter region in K-R cells.

In order to confirm the binding regions of HOTAIRM1 were transcriptional regulatory elements, we selected four binding site sequences located in four genes, two downregulated genes (FMNL2 and MACROD1) and two upregulated genes (PRDM5 and ARHGAP18) after HOTAIRM1 knockdown (Supplementary Fig. S4C), subcloned each to the upstream of a heterologous SV40 promoter in a pGL3 luciferase vector (Supplementary Fig. S4D). The luciferase activity of these constructs was then compared with that of the SV40 promoter alone after transient transfection into K-R cells. Two sequences from HOTAIRM1 binding sites in the FMNL2 and MACROD1 increased the luciferase activities, while the other two binding sequences from PRDM5 and ARHGAP18 repressed the luciferase activities (Fig. 4H). These results indicated that HOTAIRM1 binding sites played a role in transcriptional regulation, which could operate as either transcriptional enhancers or repressors.

### HOTAIRM1 affected RHOA/ROCK1 pathway by regulating ARHGAP18

**Fig. 5:**
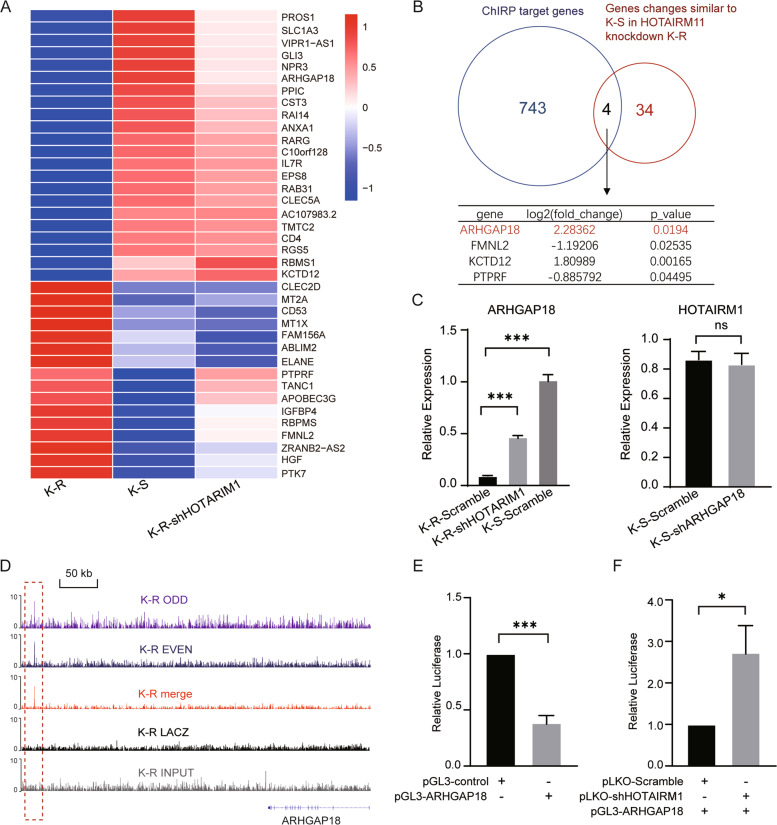
ARHGAP18 was suppressed by HOTAIRM1 binding. **A** Heatmap of the genes expression level, which may be regulated by HOTAIRM1 and contribute to GC resistance. **B** ARHGAP18 were selected by overlapping the ChIRP target genes and the genes, which became similar to K-S after HOTAIM1 knockdown in K-R. **C** The expression levels of ARHGAP18 were measured in K-S-scramble, K-R-scramble, and K-R with HOTAIRM1 knockdown using qRT-PCR (left). HOTAIRM1 was not affected by the knockdown of ARHGAP18 in K-S (right). **D** Tracks of the HOTAIRM1 binding peaks near the ARHGAP18 gene locus in ChIRP-seq data. The dark blue represents the even probe group, the purple represents the odd probe group, the red represents the peak of the merged odd and even probe data, the gray represents the LacZ probe group, and the black represents the input, the red dashed box represents the binding peak. **E** K-R cells were transfected with the HOTAIRM1 binding sequence of ARHGAP18 in luciferase reporter assay. Luciferase activity was compared with that of the empty construct with pGL3 promoter. **F** pGL3-ARHGAP18 were co-transfected into K-R cells with either nontargeting control vectors or HOTAIRM1-targeting shRNA vectors. Data information: Data were represented as mean ± SD, *n* = 3 (**C, E–F**). Difference between groups was assessed using Student’s *t*-test: *p < 0.05, **p < 0.01, ***p < 0.001, ns means no statistical significance.

In order to further find the target genes of HOTAIRM1 that affect GC resistance, we screened out 38 genes which became similar to K-S after HOTAIRM1 knockdown in K-R. (Fig. 5A). We overlapped these 38 genes with 747 ChIRP target genes (Supplementary Table S4) to obtain four intersection genes (ARHGAP18, FMNL2, KCTD12, and PTPRF) (Fig. 5B). ARHGAP18 was highly enriched in K-S and was significantly upregulated in K-R after HOTAIRM1 knockdown, while ARHGAP18 knockdown did not affect the expression of HOTAIRM1 (Fig. 5C). Moreover, ARHGAP18 was inhibited by HOTAIRM1 binding, and its binding site was regarded as a repressor (Fig. 5D, E). Next, we investigated the dependence of the transcriptional activity of ARHGAP18 repressor element on HOTAIRM1. To achieve this, the luciferase reporters were co-transfected into K-R cells along with either a nontargeting control or a HOTAIRM1-targeting shRNA vector. Remarkably, knockdown of HOTAIRM1 suppressed the function of HOTAIRM1 binding sequence of ARHAGP18 as a transcriptional repressor (Fig. 5F). Therefore, ARHGAP18 was the target gene of HOTAIRM1 and could be inhibited by HOTAIRM1.

**Fig. 6:**
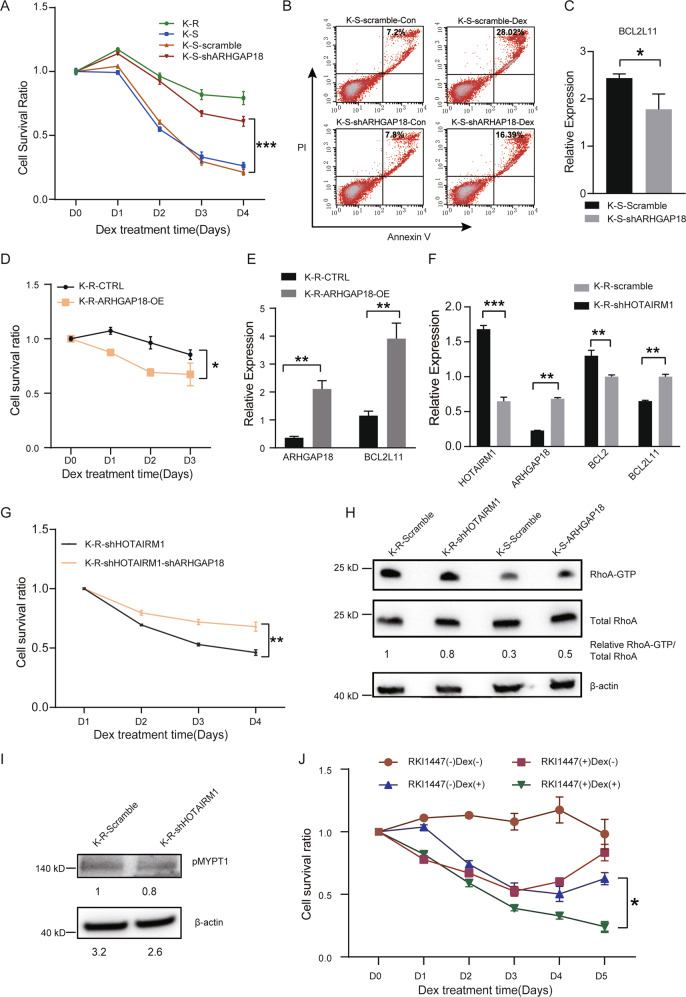
ARHGAP18 increased GC sensitivity by RHOA/ROCK1 pathway. **A** Cell viability rate in control or ARHGAP18 knockdown K-S cells after Dex (10 µM) treatment from day 0 to day 4. **B** Flow cytometry analysis of Annexin V and PI in control or ARHGAP18 knockdown K-S cells. Both cells were treated with vehicle and 10 µM dexamethasone for 48 h prior to staining. **C** Relative expression of BCL2L11 in control or ARHGAP18 knockdown K-S cells. **D** Cell viability rate in control and ARHGAP18 overexpression K-R cells after Dex (10 µM) treatment from day 0 to day 3. **E** Relative expression of BCL2L11 in control or ARHGAP18 overexpression K-R cells. **F** Relative expression of ARHGAP18, BCL2, and BCL2L11 in control or HOTAIRM1 knockdown K-R cells. **G** Cell viability rate after Dex (10 µM) treatment from day 0 to day 4. HOTAIRM1 knockdown K-R cells decreased gradually to 50% from day 0 to day 4. ARHGAP18 knockdown rescued the decrease of GC resistance caused by the knockdown of HOTAIRM1. **H** Western blot showing RhoA-GTP and total RhoA in different cells. The protein bands were analyzed in grayscale using Image J software to determine the ratio of RhoA-GTP to total RhoA. The β-actin was internal control. **I** Analysis of MYPT phosphorylated levels in control and HOTAIRM1 knockdown K-R cells. The protein bands were analyzed with Image J software to calculate the relative grayscale value of proteins between samples. The β-actin was the internal control. **J** Cell viability rate of K-R cells treated with ROCK1 inhibitors RKI-1447 (1 µM), Dex (10 µM) and combined drugs for 1–5 days, respectively. Data information: Data were represented as mean ± SD, *n* = 3 (**A, C, D–F, I**). Difference between groups was assessed using Student’s *t*-test: *p < 0.05, **p < 0.01, ***p < 0.001, ns means no statistical significance.

ARHGAP18 is a Rho GTPase-activating protein, belongs to RhoGAP family and contains classical RhoGAP domain on its C-terminus [45]. To further determine the role of ARHGAP18, we knocked down ARHGAP18 in K-S and found the cells showed stronger GC resistance than control (Fig. 6A–C). On the contrary, when ARHGAP18 was overexpressed in K-R, the cell survival rate decreased significantly after dexamethasone treatment (Fig. 6D), accompanied with a significant increase of BCL2L11 (Fig. 6E). After HOTAIRM1 knockdown in K-R, the expression of ARHGAP18 upregulated and the apoptosis-related gene (BCL2, BCL2L11) changed correspondingly (Fig. 6F). To investigate the role of HOTAIRM1- ARHGAP18 axis in glucocorticoid resistance, we knocked down ARHGAP18 in K-R-KD cells, and found that ARHGAP18 knockdown could rescued the decreased GC resistance in K-R-KD cells (Fig. 6G). The above results showed that ARHGAP18 could promote apoptosis. High expression of HOTAIRM1 inhibited ARHGAP18 and reduced the expression of BCL2L11, which conferred antiapoptotic ability on K-R cells.

As a member of RhoGAP family genes, knockdown of ARHGAP18 in K-S significantly activate RhoA (shown as RhoA-GTP) (Fig. 6H). In K-R-KD cells, the ratio of active RHOA-GTP to total RHOA decreased (Fig. 6H), and the phosphorylation level of myosin phosphatase (pMYPT1), which reflects the activity of the downstream target protein ROCK1, also decreased (Fig. 6I), indicating that RHOA/ROCK1 pathway was inhibited by HOTAIRM1 knockdown. ROCK1 inhibitor RKI-1447 could remarkably restore the sensitivity of K-R to GC (Fig. 6J). These results indicated that lncRNA HOTAIRM1 could activate RHOA/ROCK1 pathway through suppressing ARHGAP18, thereby enhancing the resistance of leukemic cells to GC-induced apoptosis.

### AML1 bound to the regulatory regions of HOTAIRM1 and ARHGAP18

**Fig. 7:**
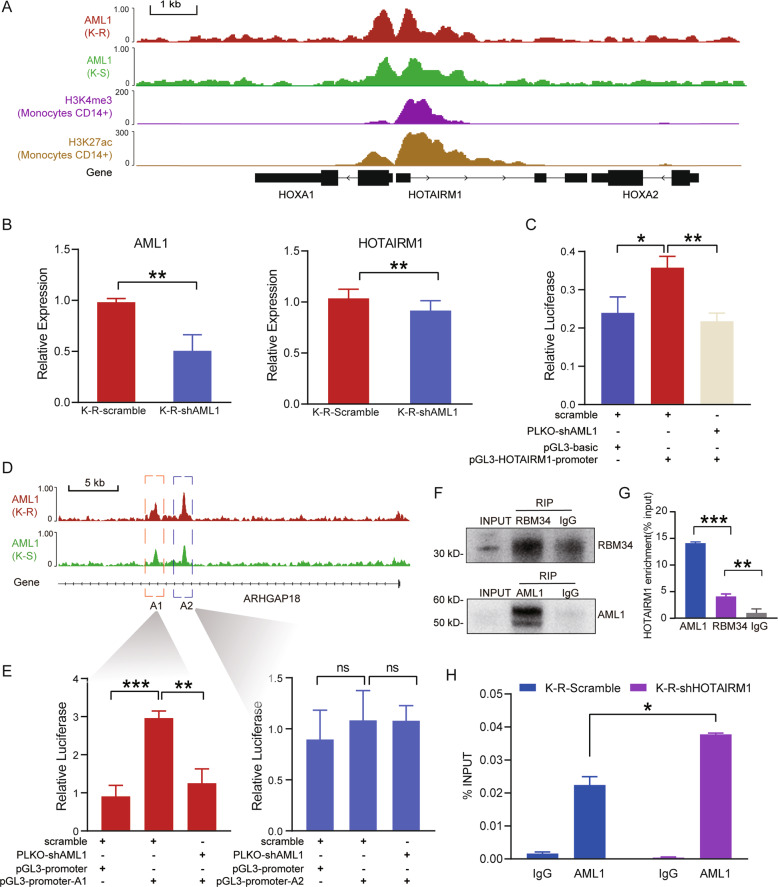
Interaction between AML1 and HOTAIRM1. **A** Tracks of binding peaks in HOTAIRM1 gene locus in various ChIP-seq data. The binding signals of AML1 in K-R and K-S cells were shown in red and green, respectively. The binding signals of H3K27ac and H3K4me3 in CD14+ monocytes were shown in purple and orange, respectively. **B** Validation of AML1 knockdown efficiency in AML1 knockdown K-R cells by qRT-PCR (left). Relative expression levels of HOTAIRM1 in control and AML1 knockdown K-R cells by qRT-PCR (right). **C** Luciferase activities were detected in control and AML1 knockdown K-R cells to confirm the binding of AML to HOTAIRM1 promoter region and the transcription regulatory activity of AML1. **D** Tracks of two AML1 binding peaks in the ARHGAP18 intron region, labeled A1 and A2. **E** Luciferase activities were detected in control and AML1 knockdown K-R cells to compare the transcriptional activity of A1 and A2 fragments and determine whether they were AML1 dependent. A1 caused enhanced luciferase activity compared with empty vector with pGL3 promoter, and the activity decreased after AML1 knockdown. A2 did not significantly change luciferase activity compared with empty vector and remained almost unchanged after AML1 knockdown. **F** Using RBM34 and AML1 antibodies to pull-down protein complexes in RIP assay. Western blot was performed with corresponding antibodies to determine the efficiency of pull-down. **G** RNAs extracted from the complexes pulled down by RBM34, AML1, and IgG antibodies were subjected to qPCR to detect the enrichment of HOTAIRM1 relative to the input (RBM34 as the positive control, IgG as the negative control). **H** ChIP was performed with AML1 antibody in control and HOTAIRM1 knockdown K-R cells. The A1 fragment bound by AML1 on ARHGAP18 was detected by qPCR to calculate the enrichment ratio relative to the input. Data information: Data were represented as mean ± SD, *n* = 3 (**B–C, E, G–H**). Difference between groups was assessed using Student’s *t*-test: *p < 0.05, **p < 0.01, ***p < 0.001, ns means no statistical significance.

Next, we studied the upstream regulation of HOTAIRM1. Because of the myeloid-specific expression of HOTAIRM1 in the previous study [46], we utilized the ChIP-seq data of myeloid-derived CD14+ monocytes, H3K4me3 (GEO: GSM1003536) and H3K27ac (GEO: GSM1003559) generated by the ENCODE Project Consortium for analysis [47]. A 1508-bp region enriched H3K4me3 and H3K27ac was found around HOTAIRM1 transcription start site (TSS) (chr7:27095336–27096843) (Fig. 7A), which was considered as a promoter to predict transcription factors. According to JASPR database [48], we found seven putative binding sites for AML1(Acute myeloid leukemia 1). It has been reported that abnormal expression of AML1 could enhance lymphocytes’ response to glucocorticoid-induced apoptosis, but the underlying reason remains unclear [49].

Furthermore, we performed chromatin-immunoprecipitation and revealed that AML1 bound to about 500 bp upstream to 1200 bp downstream of the TSS of HOTAIRM1 (Fig. 7A), which coincided with H3K4me3 and H3K27ac binding sites on HOTAIRM1. We also found the expression of HOTAIRM1 was reduced after AML1 knockdown in K-R (Fig. 7B). To further investigate whether AML1 transactivates the regulatory region of HOTAIRM1 through the above AML1 binding site, we cloned the binding site sequence into pGL3-basic luciferase reporter vector, and observed that the fragment could significantly enhance luciferase activity (Fig. 7C), confirming that this region was a transcriptional activation element. Furthermore, we knocked down AML1 in K-R and found a significant decrease in luciferase activity (Fig. 7C). These results indicated that AML1 could transactivate the HOTAIRM1 by binding to its promoter.

Analysis of ChIP-seq data of AML1 in K-R and K-S also revealed that there were two peaks in the first intron of ARHGAP18, namely A1 and A2 (Fig. 7D), which were inserted into luciferase reporter vector containing SV40 promoter respectively to detect its transcriptional activity. Compared with the empty vector, the luciferase activity of the vector with A1 fragment increased about three times, while the activity of the vector with A2 was almost unchanged. After AML1 knockdown, the luciferase activity of the vector with A1 fragment decreased to nearly empty vector level, while the luciferase activity of the vector with A2 remained unchanged (Fig. 7E). These results showed that the A1 region in ARHGAP18, rather than the A2, was a transcriptional activation element and could be activated by AML1 binding.

### Interaction of HOTAIRM1 and AML1

LncRNAs usually form complexes with proteins. We performed RNA immunoprecipitation assay (RIP) to explore whether AML1 directly interacted with HOTAIRM1. RBM34 protein was used as a positive control, since it contains two subdomains of RNA recognition motifs that can bind to RNA. We incubated the K-R cell lysate with magnetic beads coated with RBM34, AML1, and IgG protein antibodies, eluted the protein-bound RNA and detected HOTAIRM1 by qPCR. We found HOTAIRM1 was highly enriched in AML1 binding RNAs, indicating AML1 could strongly bind to lncRNA HOTAIRM1(Fig. 7F, G).

Next, we wanted to explore whether the interaction of HOTAIRM1 and AML1 could affect the transcription factor activity of AML1. We performed ChIP-qPCR on the A1 fragment of ARHGAP18 with AML1 antibody in K-R cells, and found that after HOTAIRM1 knockdown in K-R, the binding of AML1 to A1 increased (Fig. 7H). Since A1 was a transcriptional activation element and could be activated by AML1 binding, the increased binding of AML1 to A1 caused by HOTAIRM1 knockdown enhanced the transcription of ARHGAP18. The above results suggested that the interaction of HOTAIRM1 and AML1 attenuates the binding of AML1 to the A1 region of ARHGAP18 gene, thereby reducing the transcriptional activation of ARHGAP18 by AML1.

**Fig. 8:**
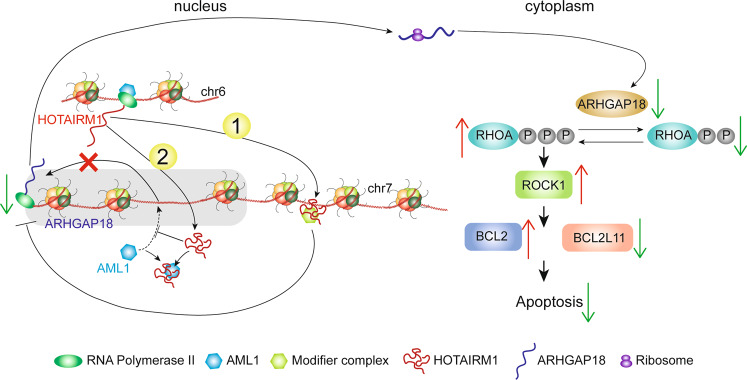
Schematic diagram of HOTAIRM1 working mechanism. The transcription of HOTAIRM1 is regulated by the binding of AML1 to its promoter region. As a trans factor, HOTAIRM1 inhibits the transcription of ARHGAP18 in the nucleus in two ways: (1) It binds to the chromatin site downstream of ARHGAP18 gene to inhibit the transcription of ARHGAP18. HOTAIRM1 may interact with other proteins to form a complex. (2) It binds to the AML1 protein to keep AML1 away from its target chromatin region, thereby preventing the gene transcription activity of AML1. After the transcription of ARHGAP18 is suppressed, its protein expression level is decreased, which increases the active form of RhoA-GTP and activates the RHOA/ROCK1 signaling pathway. Subsequently, the increase of the antiapoptotic gene BCL2 and the decrease of the proapoptotic gene BCL2L11 eventually lead to the decrease of apoptosis.

Based on all our findings, we summarized the mechanism of HOTAIRM1 on GC resistance in Fig. 8 (Fig. 8).

## Discussion

The previous studies on the mechanism of GC resistance focused mostly on GC-GR. However, GR mutations or aberrant expression were absent in many GC resistance cases, suggesting there are other unknown reasons. In our study, we constructed a cell model to simulate the occurrence of GC resistance in vitro. Based on this model, we found that lncRNA HOTAIRM1 was upregulated in GC-resistant K-R cells and activated RHOA/ROCK1 signaling pathway to resist GC-induced apoptosis, while two lncRNAs GAS5 and SRA, which were reported to be related to GC resistance by activating the upstream GR, had no significant difference between K-R and K-S. Our study reported for the first time that a lncRNA can directly activate the downstream antiapoptotic pathways, and make cells develop GC resistance. This finding reflected the specificity of lncRNA function in different systems and the complexity and heterogeneity of the causes of GC resistance, which helps us to understand the molecular mechanism of GC resistance more comprehensively. In addition, HOTAIRM1 may also be used as a marker to monitor the occurrence of GC resistance and a potential target to reverse GC resistance.

RHOA/ROCK1 signaling pathway plays a vital role in cell survival and is closely related to the pathogenesis of various diseases. When glucocorticoids are used, targeted inhibition of this pathway may help optimize glucocorticoid therapy. In addition, the influence of this pathway on cell apoptosis under other conditions or drugs needs further study.

HOTAIRM1 was discovered as a myeloid-specific long noncoding RNA in promyelocytic leukemia cell [46], which was upregulated during differentiation induced by all-trans-retinoic acid [39]. In this study, we revealed the antiapoptotic effect of HOTAIRM1 in GC resistance and explored its mechanism. So far HOTAIRM1 has been reported to play various roles in many types of cancers, including non-small cell lung cancer, glioma, colorectal cancer, endometrial cancer, ovarian cancer, etc., but most of the mechanisms are not clear [50, 51, 38, 52]. LncRNAs can interact with DNA, RNA or protein, and regulate gene expression in close proximity (*cis*-acting regulation) or target distant transcriptional activators or repressors (*trans*-acting) [53–56]. We obtained genome-wide binding sites of HOTAIRM1 by RNA immunoprecipitation for the first time, and further confirmed HOTAIRM1 could function in trans. However, HOTAIRM1 binding to chromatin to inhibit ARHGAP18 transcription may require other proteins, which need further investigation.

It has been reported that lncRNAs can bind and sequester transcription factors away from their target chromatin regions. For example, PANDA inhibited the expression of apoptotic genes by sequestering the transcription factor NF-YA from occupying target gene promoters [57]. In our study, HOTAIRM1 could interact with the AML1 protein and hinder the positive regulation of AML1 on ARHGAP18 transcription.

In addition, we found HOTAIRM1 was regulated by AML1, which is another upstream regulator after PU.1 was reported to regulate HOTAIRM1 [58]. AML1 can enhance the response of lymphocytes to glucocorticoids, but the reason is unclear [49]. Our study detailed the intermediate regulation of AML1 to GC resistance. Moreover, AML1 inhibited its own activation of ARHGAP18 by increasing the expression of HOTAIRM1, which fully demonstrates the complex and delicate regulation of lncRNA involved in eukaryotic systems.

In conclusion, our study found that long noncoding RNA HOTAIRM1 can resist GC-induced apoptosis, and elucidated the mechanism from the perspective of upstream and downstream regulation of HOTAIRM1, revealing an epigenetic cause of glucocorticoid resistance in leukemia.

## Materials and methods

### Cell culture

Human leukemia cell lines: K562, U937, THP1, Jurkat, RS4;11, and HEK-293 T cells were purchased from American Type Culture Collection (ATCC, Manassas, VA, USA.). Kasumi-1 and SKNO-1 were gifted by Dr. Janet D. Rowley from University of Chicago. CEM-C1-15, CEM-C7-14 were gifted by Dr. Rheem D. Medh from California State University. Cell lines have been authenticated. Kasumi-1 cells were cultured in complete RPMI 1640 medium containing 20% fetal bovine serum (FBS) (Gibco, Massachusetts, USA) and 1% penicillin/streptomycin at 37 °C. SKNO-1, K562, U937, THP1, CEM-C1-15, CEM-C7-14, Jurkat, RS4;11 cells were cultured in complete RPMI 1640 medium with 10% FBS, and 1% penicillin/ streptomycin at 37 °C. HEK-293 T cells were cultured in DMEM containing 10% FBS. All cells were cultured in a humidified atmosphere containing 5% CO_2_ at 37 °C, and was tested no mycoplasma contamination.

### Generation of Glucocorticoid-resistant cell lines

Equal amounts of Kasumi-1 cells (5 × 10^6^ cells) in exponential growth (~1.5 × 10^6^ cells/ml) were collected and centrifuged at 1000 rpm (~120 × *g*) for 5 min. Cell pellets were resuspended in 15 ml complete medium with either 1 μM Dex, or 0.1% EtOH (v/v) as vehicle control. Cells were then seeded into two T75 flasks with an initial cell density of ~3×10^5^ cells/ml. Most of the Dex treated Kasumi-1 cells were died post the first wave of treatment (3 days). Scarcely any change, however, could be seen in those EtOH treated cells. Therefore, Dex treated cells were all collected by centrifugation as mentioned above, and were resuspended in 15 ml fresh medium with 1 μM Dex. During the first four rounds (total 12 days), all Dex treated cells were collected, while the EtOH treated cells were normally maintained. From the fifth to seventh round, cells became less sensitive to the inhibition effect of Dex, hence subcultured every 3 days with a subcultivation ratio of 1:3. After screening of the Kasumi-1 cells for 21 days (seven rounds) altogether, both Dex and EtOH treated cells were transferred into complete medium and normally maintained for another 25 days. GC sensitivity of the Dex treated cells was analyzed by MTS-based cytotoxicity assay on the 13th, 16th, and 25th day upon withdrawal of Dex. EtOH and Dex treated cells were then defined as K-S (Kasumi-1 Sensitive) and K-R (Kasumi-1 Resistant) cells, respectively.

### Plasmids, reagents, quantitative PCR, and western blotting

HOTAIRM1 sequence was amplified by PCR (polymerase chain reaction), and then subsequently cloned into PCDH-CMV-MCS-EF1-PURO (System Biosciences, Palo Alto, CA) at EcoR I and BamH I sites. The HOTAIRM1 shRNA hairpins were cloned into pLKO lentivirus system. All shRNA oligo sequences were listed in Supplementary Table S5. High fidelity enzyme Phusion was used for PCR amplification, primers of gene overexpression were listed in Supplementary Table S6. All PCR products were verified by DNA sequencing. Specific antibodies used in this work, including mouse GR (sc-393232) (Santa Cruz Biotechnology, Texas, USA), rabbit AML1(#4334) (Cell Signaling Technology, Massachusetts, USA), mouse β-actin (M1210-2) (HangZhou HuaAn Biotechnology, China), rabbit Phospho-MYPT1 (Thr853) (#4563) (Cell Signaling Technology, Massachusetts, USA), rabbit RhoA (ARH04) (Cytoskeleton, Denver, USA). Other reagents were used include RU486 (E8875) (Sigma–Aldrich, China) and RKI-1447 (H6278) (Sigma–Aldrich, China), puromycine (A11138-03) (Gibco, Massachusetts, USA). All qPCR primers were designed with Primer 5.0 and synthesized by Sangon Biotech (Beijing, China) (Supplementary Table S7, S8). qPCR was performed using SYBR Green PCR Master Mix (#kk4601) (KAPA Biosystems, Woburn, USA) by Two-step method for three technical replicates, and the significance was determined by two-tailed *t*-test. Each experiment was repeated at least three times independently. Western blotting was carried out with standard protocol. The band intensities on western blot were quantified with Image J and each control was normalized to obtain the ratio of protein level.

### Subcellular fractionation

Fractionation of the cells to separate nucleus and cytoplasm was performed using NE-PER Nuclear and Cytoplasmic Extraction Reagents (#78833) (Thermo Scientific, Massachusetts, USA) following the manufacturer’s protocol. The separation efficiency of the two fractions was assessed by qPCR amplification with primers targeting the nucleus-specific U1 and cytoplasmic specific GAPDH.

### Chromatin-immunoprecipitation (ChIP)

K-R and K-S cell were exposed to 10^−6^ M of dexamethasone or corresponding vehicle ethanol for 8 h. They were then fixed by formaldehyde with 1% final concentration and incubated for 10 min at room temperature with rotation. The crosslinking reactions were quenched with 0.125 M glycine for 5 min at room temperature and washed twice with ice-cold PBS (phosphate buffer saline). And ChIP was performed by co-precipitating the DNA/protein complexes with a GR antibody (sc-393232) (Santa Cruz Biotechnology, Texas, USA) and a rabbit control IgG (Santa Cruz Biotechnology, Texas, USA). DNA from protein-associated complexes and corresponding input samples was recovered and purified using QIAquick PCR Purification Kit (QIAGEN, Hilden, Germany). For ChIP-seq, the ChIP DNA samples were dissolved in 20 μl water and sequenced with a 20 M reads per sample using Hiseq2500 platform.

### Construction and transduction of shRNA-expressing lentiviral vector

The shRNA-targeting sites (21 nt) of HOTAIRM1 transcript were selected on the shRNA selection server of the Whitehead Institute for Biomedical Research, and their specificities were detected by BLAST. Lentiviral vectors expressing short hairpin RNAs (shRNAs) targeting the selected HOTAIRM1 sites (Supplementary Table S5) were constructed by inserting each shRNA cassette into a pLKO-TRC backbone plasmid (Addgene plasmid 10878). A control vector expressing a scramble shRNA sequence (adopted from Addgene plasmid 1864) was constructed on the same backbone plasmid. Resultant pLKO.1-TRC-shHOTAIRM1 and control pLKO-TRC scramble shRNA lentiviral vectors were prepared and pseudotyped in the HEK293T cell line by co-transfection with packaging plasmids pMD2.G (Addgene plasmid 12259) and psPAX2 (Addgene plasmid 12260) using FuGENE® 6 Transfection Reagent (Promega, Madison, Wisconsin, USA). For transduction, K-R cells were suspended in a medium containing lentiviral supernatant and cultured at 37 °C for 48–72 h. For generation of stably transduced cell clones, the transgenic cells were inoculated in puromycin-containing medium for 5–7 days, and showed HOTAIRM1 knockdown rate of over 70% by qRT-PCR.

### RNA-seq

Total RNAs were isolated using TRIzol reagent (Invitrogen, Shanghai, China). Construction of ribosomal removal transcriptome library and RNA-seq was carried out using Illumina HiSeq2500 (Novo gene, Beijing, China).

### Chromatin isolation by RNA purification (ChIRP-seq) and analysis

The ChIRP procedure was performed as previously described [41]. DNA probes (Supplementary Table S9) with 20 bp biotinylated at the 3’ end were used (Sangon Biotech, Beijing, China). About 1 × 10^8^ K-R cells were harvested and washed twice with frozen PBS. Then, cells were resuspended in 4 ml PBS. For chemical crosslinking, cells were fixed on plate with appropriate amounts of 3% formaldehyde in PBS for 30 min at room temperature. Crosslinking was then quenched with 0.125 M glycine for 5 min. Cells were rinsed again with PBS, and pelleted at 800 × *g* for 5 min, followed by two times of frozen PBS wash. Crosslinked cell pellets were resuspended in lysis buffer (50 mM Tris-Cl, pH 7.5, 10 mM EDTA, 1% SDS) with addition of protease inhibitor cocktail, PMSF and RNaseOut. Chromatin fractions were sonicated to have average DNA size ranging from 200–1000 bp. The supernatant was saved for probe hybridization. For each hybridization reaction, about 10 pmol of biotinylated probes were used. About 1/4 volume of 5× Hybridization buffer (50 mM Tris-Cl, pH 7.5, 10 mM EDTA, 1% SDS, 1.5 M NaCl, 50% formamide), was added and incubated at 37 °C by end-to-end rotation overnight. Then, 50 μl of prebalanced streptavidin T1 beads were added and incubated at 37 °C by end-to-end rotation overnight. The beads were then washed twice at 50 °C by high salt wash buffer (20 mM Tris-HCl, 500 mM NaCl, 1% SDS), followed by 3 washes with 0.1× SSC wash buffer (0.1× SSC, 1% SDS) at 37 °C. We allowed 15–20 min of end-to-end rotation per wash. For DNA isolation, the beads were further washed one time with SDS elution buffer (50 mM Tris-Cl, 5 mM MgCl_2_, 75 mM NaCl, 1% SDS) at 37 °C for 20 min, and one time with elution buffer (50 mM Tris-Cl, 5 mM MgCl_2_, 75 mM NaCl, 0.1% Triton X-100) at 39 °C for 5 min. Then, DNA was eluted sequentially by RNase H elution buffer (50 mM Tris-Cl, 5 mM MgCl_2_, 75 mM NaCl, 0.1% Triton X-100, 0.5U/l RNase H) at 37 °C for 20 min, and by SDS elution buffer at room temperature for 2 min. The eluents were combined. And the crosslinking was reversed in the presence of 0.1 µg/µl protease K, 150 mM NaCl, and 10 mM EDTA incubate at 50 °C. Then add 300 μl PhOH:Chloroform:Isoamyl per sample. Shake vigorously for 10 min, and spin down on a benchtop centrifuge at 16,100 × *g* for 5 min at 4 °C. Take aqueous from the top. Add 30 μl NaOAc, and 900 μl 100% EtOH. Mix well and store at −20 °C overnight. Spin samples at 16,100 × *g* for 30 min at 4 °C. Decant supernatant carefully. Add 1 mL 70% EtOH and vortex to mix. Spin at 16,100 × *g* for 5 min. Remove supernatant by pipette. Air dry for 1 min. Resuspend in 15 μl ddH_2_O. Measure DNA concentration with Quant-iT™ PicoGreen™ dsDNA Assay Kit (P7589) (Invitrogen, Shanghai, China). Library construction was done by using NEB library construction modules and following the instrument of NEBNext® ChIP-Seq Library Prep Reagent Set for Illumina (#E6200S) (California, USA).

### Sequencing data analysis

RNA-Seq reads were mapped using TopHat v2.0.11 software [59]. For K-R and K-S data, HT-Seq was used to calculate the gene counts [60], and then DEGSeq [61] was used to analyze the differentially expressed genes. For K-R-Scramble and K-R-shHOTAIRM1, FPKM (Fragments Per Kilobase per Million) values were calculated using Cufflink 2.1.1 to represent expression levels [62]. For ChIRP and DNA-Seq data, raw reads were uniquely mapped to the human genome (hg38) using Bowtie v.1.0.0 software [63]. Positive peaks were identified by MACS [43] program by comparing the wild-type and knockout-type samples with p-cutoff value of 1 × 10^−5^. ChIP-Seq reads were aligned using Bowtie, and peaks were identified using MACS v.1.4.2 software [43].

### Luciferase reporter assay

Luciferase reporter assay was performed with pGL3 vectors of Promega (Madison, Wisconsin, USA), and luminescence was detected with Dual-Glo system from Promega. HOTAIRM1 binding DNA sequences were amplified by PCR, and dsDNAs were inserted into pGL3-promoter vector between SmaI and XhoI cleavage sites, which is 1 kb downstream of 3’ end of the firefly luciferase gene. The cloned vector was co-transfected with pRL-TK renilla luciferase control reporter vectors into Kasumi-1 cells. The firefly and renilla luminescence were detected in the transfected cells after 48 h using Dual-Glo^®^luciferase Assay System (Promega, Madison, Wisconsin, USA). The firefly luminescence was normalized to renilla luminescence under various conditions. Folding inductions were then calculated by normalizing to the pGL3-promoter control.

### Cell viability assay

The cells in logarithmic growth phase were washed twice with PBS and detached by trypsin to make single-cell suspension. The cells were inoculated into a 96-well plate, 5 × 10^3^ cells per well (six duplicated wells), and incubated in 5% CO_2_ incubator for 24–72 h at 37 °C. After that, 20 μl MTS (Promega, Madison, Wisconsin, USA) solution was added to each well. The plate was incubated in 5% CO_2_ incubator at 37 °C for another 4 h. Subsequently, the solution was removed. DMSO (150 μl) were added in the walls and shaked for 10 min to dissolve the crystal. The optical density (OD) value of each well at 0, 24, 48, and 72 h was read. MTT curve graph was constructed with OD value as ordinate, and the interval time as abscissa. The experiment was repeated three times.

### Flow cytometry

The cells were centrifuged to remove the media, and then resuspended in 100 μl of Annexin V Binding Buffer (BD PharMingen, New Jersey, USA) containing 1 mg/ml propidium iodide (Sigma–Aldrich) and APC-Annexin V (BioLegend, San Diego, USA) for 15 min. Then 400 μl of Annexin V Binding Buffer (BD PharMingen, New Jersey, USA) was added to the samples. The fluorescence was measured using BD LSR II flow cytometer or Gallios flow cytometer (Beckman Coulter, California,USA), and then analyzed by Flow Logic software (Miltenyi Biotec, Germany).

### RhoA activation assay

RhoA activity was determined using RhoA Pull-down Activation Assay Kit (BK036-S, Cytoskeleton, Denver, USA).

## Supplementary information

Supplemental Fig S1

Supplemental Fig S2

Supplemental Fig S3

Supplemental Fig S4

supplementary figure legends

Supplementary Table S1

Supplementary Table S2

Supplementary Table S3

Supplementary Table S4

Supplementary Table S5

Supplementary Table S6

Supplementary Table S7

Supplementary Table S8

Supplementary Table S9

## Data Availability

The raw sequence data have been deposited in the Genome Sequence Archive in BIG Data Center, Beijing Institute of Genomics (BIG), Chinse Acadamy of Science, under accession number CRA002779 and CRA002778.
