# Supplementary figures and images for: The long noncoding RNA HOTAIRM1 controlled by AML1 enhances glucocorticoid resistance by activating RHOA/ROCK1 pathway through suppressing ARHGAP18
Source: Cell Death Dis. 2021 Jul 14;12(7):702. doi: 10.1038/s41419-021-03982-4 (PMC8280127; doi:10.1038/s41419-021-03982-4)

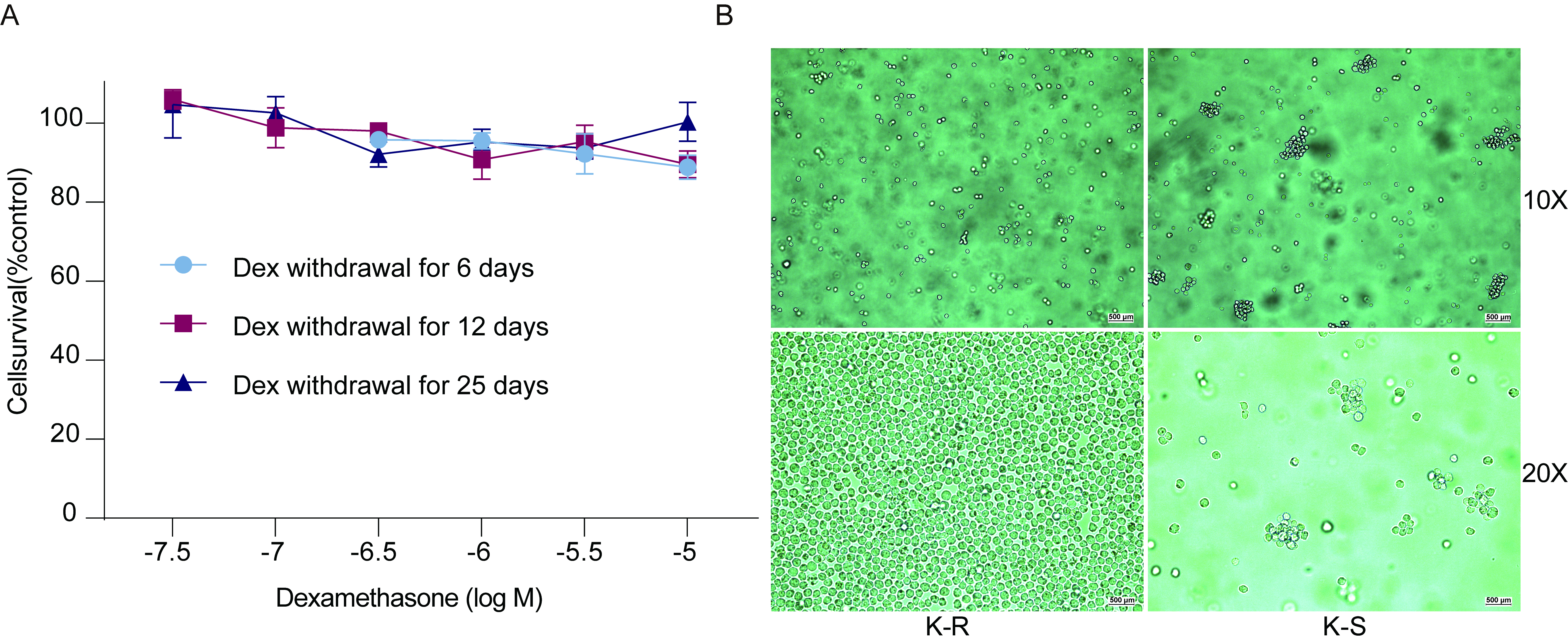

Supplement: Supplementary file 1 — Supplemental Fig S1 [file 41419_2021_3982_MOESM1_ESM.tif]

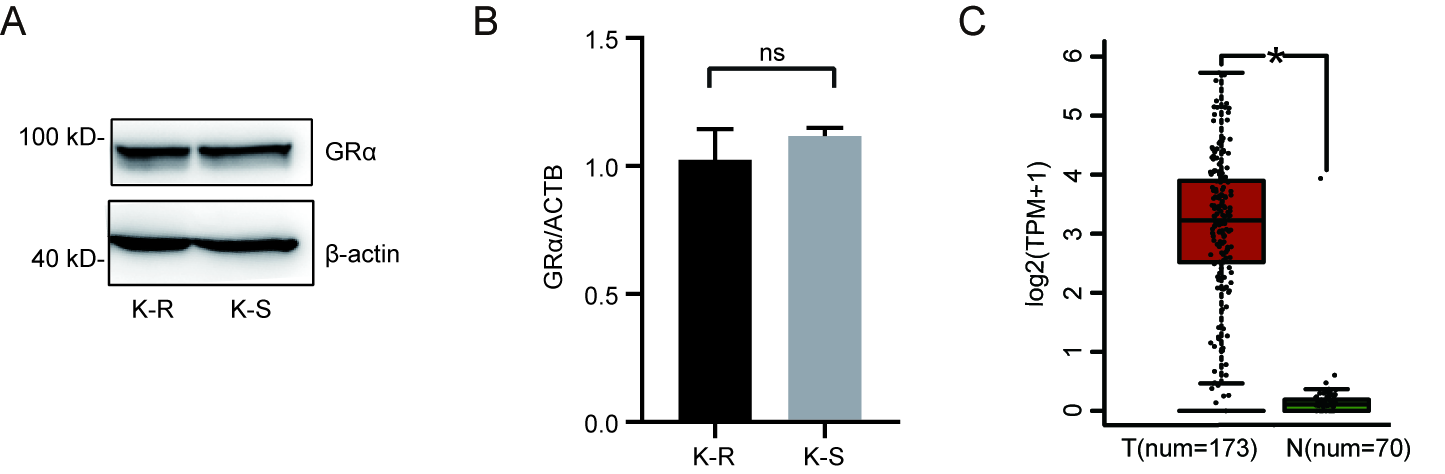

Supplement: Supplementary file 2 — Supplemental Fig S2 [file 41419_2021_3982_MOESM2_ESM.tif]

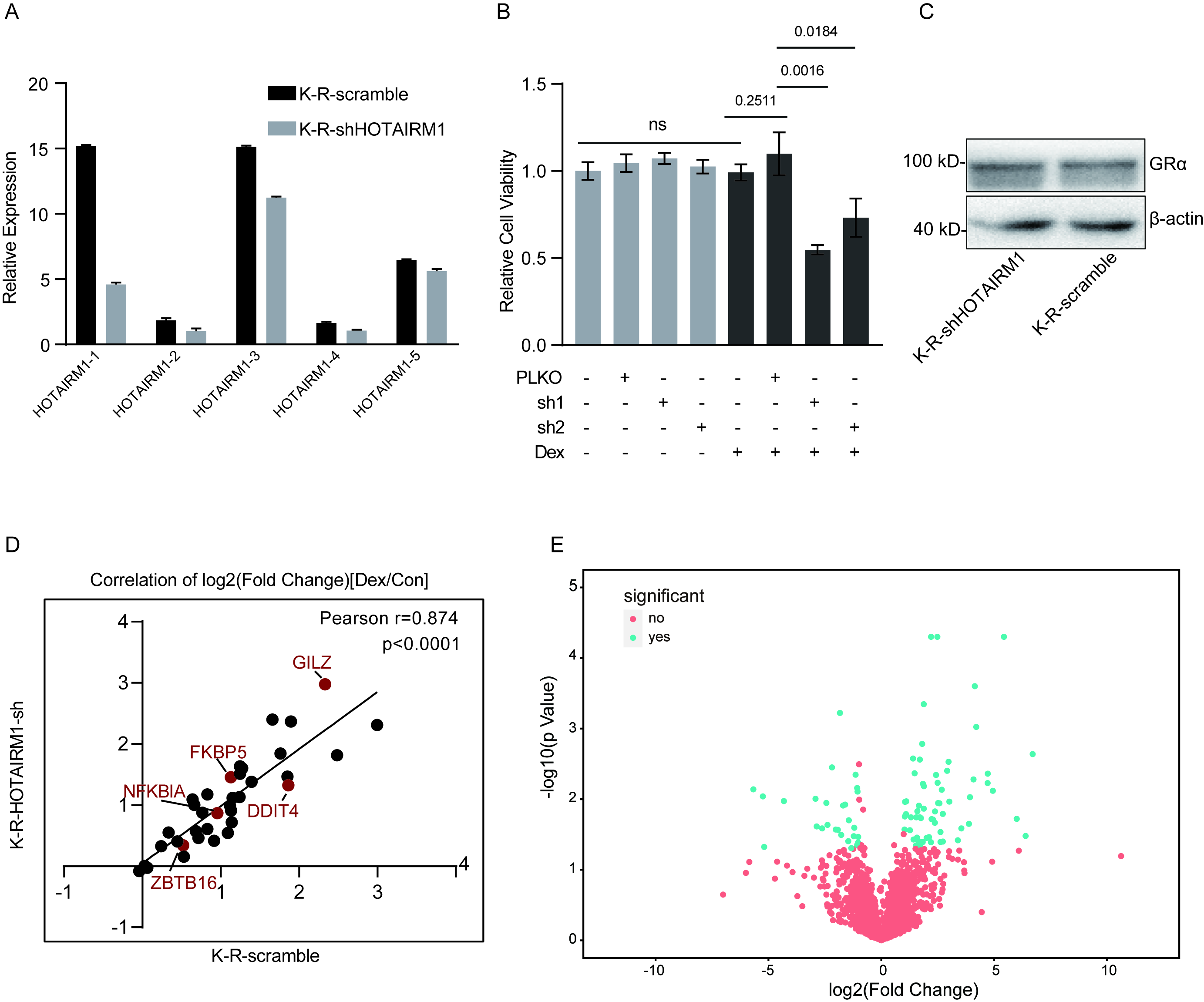

Supplement: Supplementary file 3 — Supplemental Fig S3 [file 41419_2021_3982_MOESM3_ESM.tif]

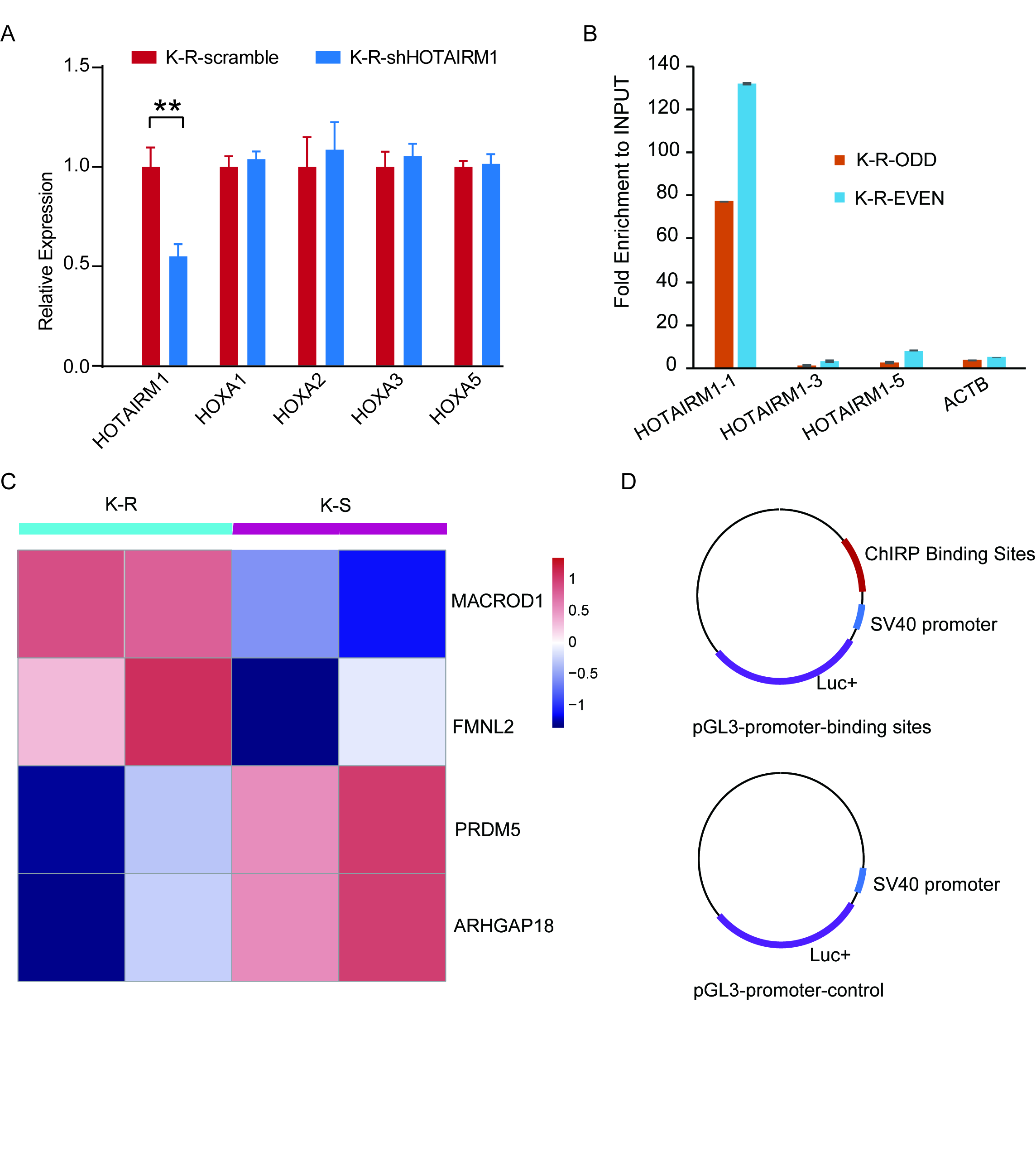

Supplement: Supplementary file 4 — Supplemental Fig S4 [file 41419_2021_3982_MOESM4_ESM.tif]
