# Supplementary material for: The long noncoding RNA HOTAIRM1 controlled by AML1 enhances glucocorticoid resistance by activating RHOA/ROCK1 pathway through suppressing ARHGAP18
Source: Cell Death Dis. 2021 Jul 14;12(7):702. doi: 10.1038/s41419-021-03982-4 (PMC8280127; doi:10.1038/s41419-021-03982-4)
Supplement: Supplementary file 5 — supplementary figure legends [file 41419_2021_3982_MOESM5_ESM.docx]

## Supplementary Figures

Figure S1:

1. K-R withdrawal culture for 13, 16, 25 days, and cells were treated with various concentrations of dexamethasone for 4 days, the cell viability was measured by the MTS assay. Data were represented as mean ± SD, n=3.
2. The morphology of K-R and K-S respectively (from left to right). Top: 10-fold magnification, Bottom: 20-fold magnification. Scale bars: 500 μm.

Figure S2:

1. GRα protein levels were analysed by Western blotting in K-R and K-S. β-actin was used as loading control.
2. GRα protein expression values normolized with β-actin in K-R and K-S. The western blot protein bands were analyzed in grayscale using Image J software. Data were represented as mean ± SD, n=3. Difference between groups was assessed using Student’s t test: *p < 0.05, **p < 0.01, ***p < 0.001, ns means no statistical significance.
3. The relative expression level of HOTAIRM in LAML patients (n=172) and healthy samples (n=70) in the TCGA database. The ordinate is the number of reads per million (TPM, Transcripts Per Million) of HOTAIRM1 by Log2(TPM+1) operation. Difference between groups was assessed using Student’s t test: *p < 0.05, **p < 0.01, ***p < 0.001, ns means no statistical significance.

Figure S3:

1. Expression levels of five variants of HOTAIRM1 in control and HOTAIRM1 knockdown K-R cells using qRT-PCR. Data were represented as mean ± SD, n=3.
2. The relative cell viability of the control group and the HOTAIRM1 gene knockdown group was measured by MTS at the 4th day with and without Dex (10^-5^ M) treatment. Data were represented as mean ± SD, n=3. Difference between groups was assessed using Student’s t test, ns means no statistical significance.
3. Knockdown of HOTAIRM1 in K-R followed by Western Blot detection of protein expression of functional glucocorticoid receptor GRα.
4. Correlation analysis of gene expression ratio of genes regulated by GR in knockdown of HOTAIRM1 and control K-R cells, red dots indicate genes indicate genes regulated by GR and playing a role in GC-induced apoptosis in previous reports.
5. Volcano map of the genes after knocking down HOTAIRM1 in K-R, green dot represents significantly different genes (Log2 Fold Change> 1 or <-1, p value < 0.05).

Figure S4:

1. Expression levels of HOXA1, HOXA2, HOXA3, HOXA5 were measured in control and HOTAIRM1-knockdown K-R cells using qRT-PCR. Data were represented as mean ± SD, n=3. Difference between groups was assessed using Student’s t test: *p < 0.05, **p < 0.01, ***p < 0.001, ns means no statistical significance.
2. Using ACTB as a reference, the enrichment degree relative to INPUT of the three main transcripts of HOTAIRM1 hybridized by odd and even probe pools was detected. Data were represented as mean ± SD, n=3, ns means no statistical significance.
3. Heatmap of four HOTAIRM1 binding genes (two genes are upregulated after HOTAIRM1 knockdown, and two are downregulated after HOTAIRM1 knockdown) differentially expressed in K-R cells with HOTAIRM1 knockdown.
4. Luciferase reporter vector diagram. The red segment represents inserted ChIRP-seq binding sites, the blue segment represents SV40 promoter, and the purple segment represents luciferase reporter gene.
